# Optimizing Daily Light Integral in Seedling Stage Accelerates Heading and Flowering in Wheat Under LED Lighting

**DOI:** 10.3390/plants15020326

**Published:** 2026-01-21

**Authors:** Luming Zhong, Xiang Ji, Jun Liu, Qing Zhou, Dongxian He

**Affiliations:** Key Laboratory of Agricultural Engineering in Structure and Environment of MOARA, College of Water Resources & Civil Engineering, China Agricultural University, Beijing 100083, China; luming@cau.edu.cn (L.Z.); jx@cau.edu.cn (X.J.); jun.liu.ag@gmail.com (J.L.); cauzhou@cau.edu.cn (Q.Z.)

**Keywords:** wheat seedling, daily light integral (DLI), speed-breeding, LED plant factory, heading

## Abstract

Conventional wheat breeding in the field is limited to two generations per year and is susceptible to environmental fluctuations. Speed-breeding in a plant factory offers a solution; however, recommendations for lighting strategies remain limited. To identify the optimal daily light integral (DLI) for the seedling stage, we tested three light intensities (300, 500, and 700 μmol m^−2^ s^−1^) and four photoperiods (10, 14, 18, and 22 h d^−1^), resulting in DLIs ranging from 10.8 to 55.4 mol m^−2^ d^−1^. Our results indicated that an optimal DLI of 39.6 mol m^−2^ d^−1^ was associated with the highest seedling index (0.26) and root-to-shoot ratio (0.42), as well as enhanced photosystem performance. Beyond this DLI, these parameters and shoot biomass plateaued as the DLI increased. Moreover, treating seedlings with this optimal DLI of 39.6 mol m^−2^ d^−1^ (using 500 μmol m^−2^ s^−1^ light intensity and a 22 h d^−1^ photoperiod) resulted in heading and flowering 5.9 and 7.5 days earlier after transplanting, respectively, than those under the lowest DLI (10.8 mol·m^−2^·d^−1^). This study established a lighting strategy to produce high-quality seedlings and accelerate heading and flowering, thereby offering a valuable physiological framework for advancing speed-breeding systems in wheat.

## 1. Introduction

Wheat (*Triticum aestivum* L.), one of the world’s major staple crops, supplies 20% of global protein and calories [1]. Its production stability is critical to global food security. It is projected that the global population will exceed 10 billion within the next 30 years [2], with total food demand expected to increase by 35–56% [3]. However, the current yield increases in major grain crops, including wheat, are failing to keep pace with future demands [4,5]. Enhancing crop yield and resilience through genetic improvement is a crucial strategy for addressing global food security challenges. Nevertheless, the rate of genetic gain in conventional wheat breeding is severely constrained by both long breeding cycles (only one to two generations per year) and frequent extreme climate events [6,7]. Therefore, utilizing light-emitting diodes (LEDs) in plant factories for speed-breeding to overcome weather constraints and shorten the growth cycle has emerged as a vital approach for accelerating breeding and achieving sustainable food production.

LED plant factories facilitate year-round crop production with precise environmental control, presenting distinct advantages for crop breeding. By replacing traditional off-site shuttle breeding, they enable 5–6 generations per year, substantially accelerating generational progress. Among all environmental factors, light plays a dual role: as the primary energy source for photosynthesis [8,9] and as a key signal regulating morphogenesis [10,11], physiological metabolism [12], and flowering and seed set [13,14]. Thus, optimizing light conditions can substantially promote crop growth and development, thereby accelerating the seed production cycle.

As a long-day plant, wheat exhibits accelerated development under an extended photoperiod. In plant factories with artificial lighting, a photoperiod of 22 h of light and 2 h of dark enables up to six generations per year for spring and durum wheat [15]. Also, super-long lighting (22 h per day) not only shortens the growth cycle in spring and semi-winter wheat varieties but also induces reproductive transition without vernalization, demonstrating a “vernalization compensation effect” [16]. Beyond photoperiod, the pattern of the light/dark cycle profoundly influences reproductive dynamics. For example, a 6 h light/6 h dark cycle accelerated heading time and spike development compared to a 12 h light/12 h dark cycle [17]. Furthermore, photoperiod interacts with light quality: supplementing red light to extend the photoperiod has been shown to shorten spike development time and increase the number of fertile florets [18].

As a key parameter integrating light intensity and photoperiod, the daily light integral (DLI) represents the total photosynthetically active radiation received by plants per day and plays a significant regulatory role in crop growth and development. Studies have shown that the DLI regulates biomass accumulation by directly affecting the efficiency of photosynthetic carbon assimilation [19]. Under appropriate DLI conditions, Chinese cabbage can enhance its photosynthetic capacity by optimizing nitrogen partitioning and leaf anatomical structure [20]. Furthermore, the DLI can significantly improve water use efficiency [21] and regulate flowering time, branching morphology, and overall biomass allocation [22]. However, existing research has primarily focused on vegetable and ornamental crops, while systematic investigation into the regulatory effects and physiological mechanisms of DLI on wheat growth and development across different stages remains limited.

Seedling quality is critical for wheat performance after transplanting, as vigorous seedlings not only tend to till more but also have higher photosynthetic capacity that better supports spike development, ultimately influencing the entire growth cycle. We therefore hypothesize that co-optimizing the DLI and photoperiod during the seedling stage can enhance wheat seedling growth, improve seedling quality, and accelerate heading and flowering. This study aims to investigate the effects of different DLI levels during the seedling stage on wheat growth, photosystem development, and heading and flowering. The objective is to identify the optimal DLI combination for wheat seedlings and to elucidate its underlying physiological responses. The findings will provide a lighting protocol to improve seedling establishment and support wheat speed-breeding systems.

## 2. Results

### 2.1. Morphology of Wheat Seedlings

**Figure 1:**
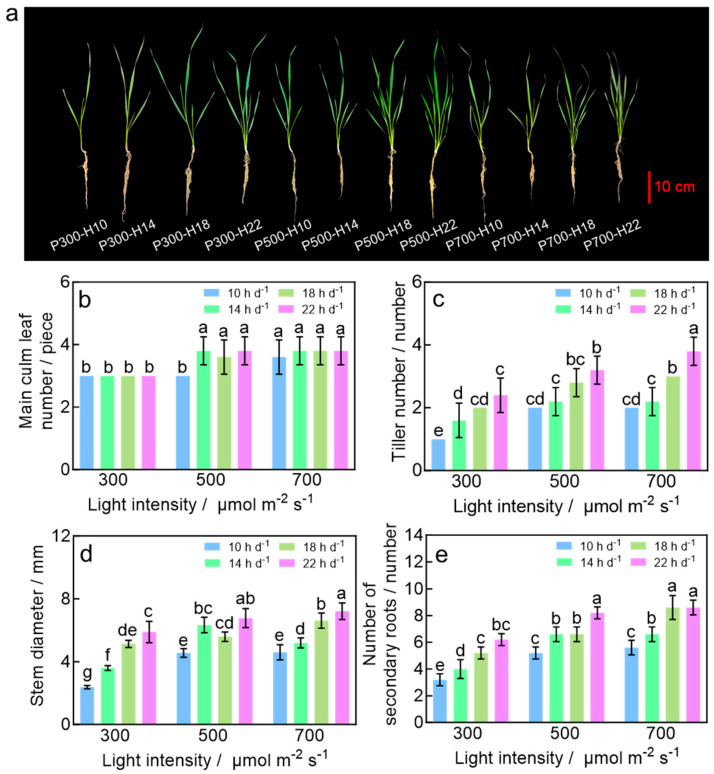
Representative photos (**a**), main culm leaf number (**b**), tiller number (**c**), stem diameter (**d**), and number of secondary roots (**e**) of wheat seedlings in response to light treatments (light intensity and photoperiod). Different letters indicate significant differences (*p* < 0.05), according to Duncan’s multiple range test.

The DLI significantly influenced the morphogenesis of wheat seedlings. The superior morphology was observed under a DLI of 39.6 mol m^−2^ d^−1^ (the P500-H22 treatment), with the main culm leaf number of 3.8 (Figure 1a,b). The P500-H22 treatment resulted in a high tiller number, which was second only to P700-H22 (corresponding to a DLI of 55.4 mol m^−2^ d^−1^, Figure 1c). Under a DLI of 39.6 mol m^−2^ d^−1^ (the P500-H22 treatment), stem diameter and number of secondary roots were among the highest, reaching 6.78 mm and 8.2, respectively (Figure 1d,e). These results demonstrate that, while tiller number was higher at the highest DLI of 55.4 mol m^−2^ d^−1^ (P700-H22 treatment), most morphological parameters of wheat seedlings peaked at or near a DLI of 39.6 mol m^−2^ d^−1^.

### 2.2. Quality of Wheat Seedlings

**Figure 2:**
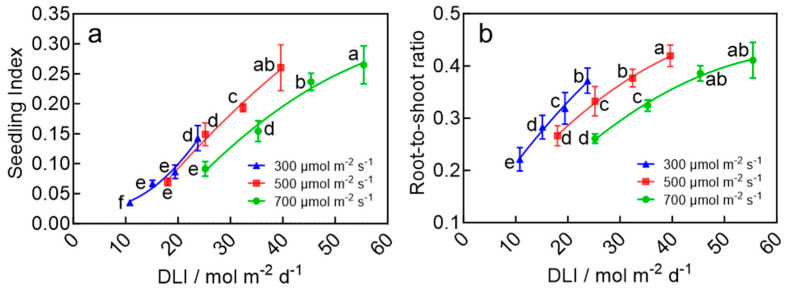
Seedling index (**a**) and root-to-shoot ratio (**b**) of wheat seedlings in response to a DLI under the LED plant factory. The four data points on each curve of the same color correspond to photoperiods of 10, 14, 18, and 22 h d^−1^, respectively, under the same light intensity. Different letters for the same parameter indicate significant differences (*p* < 0.05), according to Duncan’s multiple range test.

Seedling quality was assessed using the seedling index and root-to-shoot ratio, both of which showed strong dependence on the DLI. At a DLI of 39.6 mol m^−2^ d^−1^, the seedling index and root-to-shoot ratio reached 0.26 and 0.42, respectively (Figure 2). These values were significantly higher than those in treatments with a lower DLI and did not differ significantly from the values observed under a DLI of 45.4 and 55.4 mol m^−2^ d^−1^. Furthermore, under the same light intensity, extending the photoperiod significantly enhanced both the seedling index and root-to-shoot ratio. These findings indicate that increasing the DLI up to 39.6 mol m^−2^ d^−1^ effectively improves the robustness of wheat seedlings.

### 2.3. Biomass Accumulation of Wheat Seedlings

**Figure 3:**
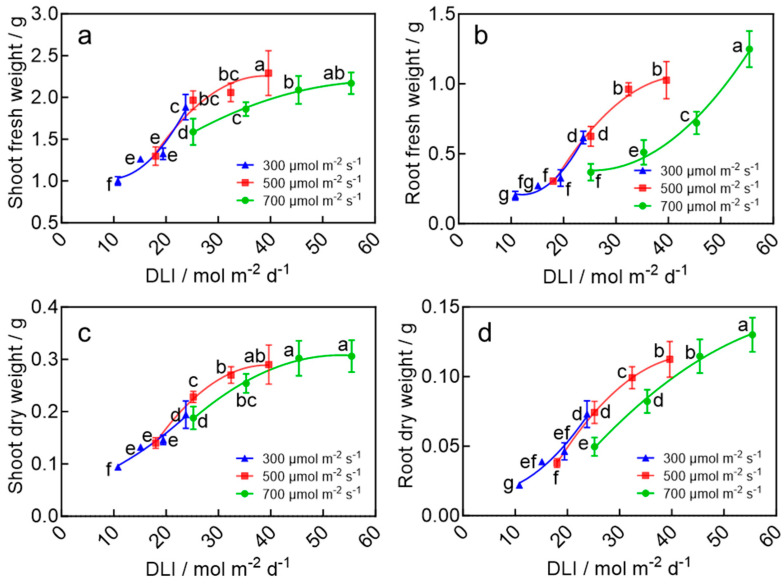
Shoot fresh weight (**a**), root fresh weight (**b**), shoot dry weight (**c**), and root dry weight (**d**) of wheat seedlings in response to a DLI under the LED plant factory. The four data points on each curve of the same color correspond to photoperiods of 10, 14, 18, and 22 h d^−1^, respectively, under the same light intensity. Different letters for the same parameter indicate significant differences (*p* < 0.05), according to Duncan’s multiple range test.

The DLI had a significant influence on biomass accumulation in wheat seedlings. Both fresh and dry weights of the shoots initially increased and then plateaued with an increasing DLI (Figure 3a,c). The maximum shoot fresh weight, 2.29 g, was observed at a DLI of 39.6 mol m^−2^ d^−1^. This value was significantly higher than those in most other treatments, except for the DLI of 55.4 mol m^−2^ d^−1^, which showed no significant difference. The maximum shoot dry weight, 0.31 g, was recorded under the DLI of 55.4 mol m^−2^ d^−1^, though it did not differ significantly from the values obtained at a DLI of 39.6 and 45.4 mol m^−2^ d^−1^. In contrast, both fresh and dry weights of the roots increased continuously with an increasing DLI, reaching their highest values of 1.25 g and 0.13 g, respectively, under the highest DLI treatment (55.4 mol m^−2^ d^−1^), which were significantly greater than all other treatments (Figure 3b,d). Additionally, under the same light intensity, extending the photoperiod significantly promoted biomass accumulation in all plant parts, with the maximum values achieved under a 22 h photoperiod. These results indicate that increasing the DLI enhances aboveground biomass accumulation up to 39.6 mol m^−2^ d^−1^, beyond which no further significant improvement is observed. In contrast, root biomass accumulation was continuously enhanced as the DLI increased.

### 2.4. Leaf Chlorophyll Contents of Wheat Seedlings

**Figure 4:**
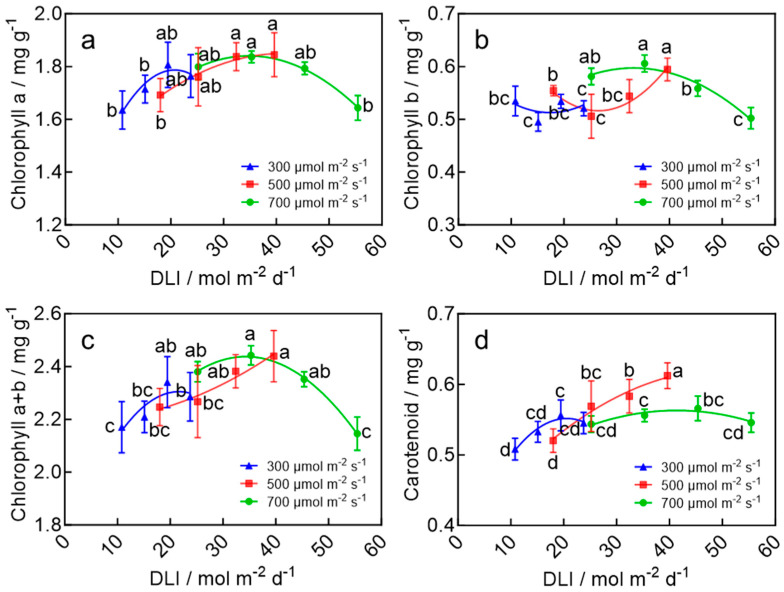
Chlorophyll a (**a**), Chlorophyll b (**b**), Chlorophyll a + b (**c**), and carotenoid (**d**) of wheat seedling leaves in response to the DLI under the LED plant factory. The four data points on each curve of the same color correspond to photoperiods of 10, 14, 18, and 22 h d^−1^, respectively, under the same light intensity. Different letters for the same parameter indicate significant differences (*p* < 0.05), according to Duncan’s multiple range test.

The chlorophyll content in wheat seedling leaves was significantly influenced by the DLI. As the DLI increased, the contents of chlorophyll a, chlorophyll b, total chlorophylls (a + b), and carotenoids all exhibited an initial increase followed by a decline (Figure 4). Specifically, chlorophyll a and total chlorophylls reached their maximum values of 1.84 mg g^−1^ and 2.44 mg g^−1^, respectively, at a DLI of 39.6 mol m^−2^ d^−1^ (Figure 4a,c). Similarly, carotenoid content reached a peak of 0.61 mg g^−1^ under the same DLI, which was significantly higher than other treatments (Figure 4d). In contrast, chlorophyll b content reached its maximum value of 0.61 mg g^−1^ at a slightly lower DLI of 35.3 mol m^−2^ d^−1^, showing no significant difference compared to the values at a DLI of 25.2 and 39.6 mol m^−2^ d^−1^, but was significantly higher than all other treatments (Figure 4b). These findings indicate that a DLI within the range of approximately 35–40 mol m^−2^ d^−1^ is suitable to maximize the synthesis and accumulation of photosynthetic pigments in wheat seedling leaves.

### 2.5. Leaf Chlorophyll Fluorescence of Wheat Seedlings

**Figure 5:**
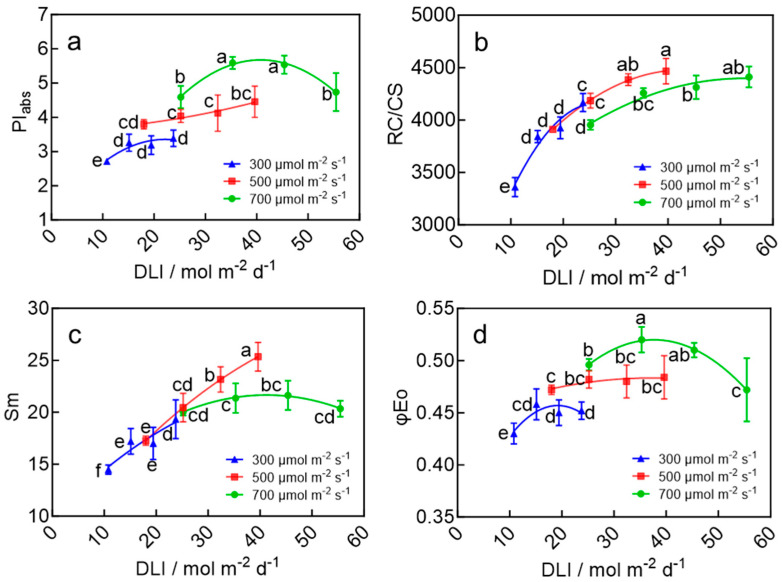
PI abs (**a**), RC/CS (**b**), Sm (**c**), and φEo (**d**) of wheat seedling leaves in response to the DLI under the LED plant factory. The four data points on each curve of the same color correspond to photoperiods of 10, 14, 18, and 22 h d^−1^, respectively, under the same light intensity. Different letters for the same parameter indicate significant differences (*p* < 0.05), according to Duncan’s multiple range test.

The DLI significantly affected the development of the photosynthetic apparatus in wheat seedling leaves. Both the photosynthetic performance index (PIabs) and the quantum yield for electron transport (φEo) exhibited an initial increase followed by a decrease with a rising DLI, reaching maximum values of 5.59 and 0.52, respectively, at a DLI of 35.3 mol m^−2^ d^−1^ (Figure 5a,d). These values were significantly higher than those in other treatments, except for the DLI of 45.4 mol m^−2^ d^−1^, which showed no significant difference (Figure 5a,d). Similarly, the pool size of electron carriers on the acceptor side of PSII (Sm) showed a trend of initial increase and subsequent decrease, peaking at 25.34 under a DLI of 39.6 mol m^−2^ d^−1^, which was higher than all other treatments (Figure 5c). In contrast, the density of active reaction centers per cross-sectional leaf area (RC/CS) increased initially and then stabilized with an increasing DLI, reaching a maximum of 4466.5 at a DLI of 39.6 mol m^−2^ d^−1^—a significant increase of 32.9% compared to the lowest DLI treatment (Figure 5b). Furthermore, with the increase in the DLI, the energy absorbed (ABS/CSo), trapped (TRO/CSo), transferred (ETO/CSo), and dissipated (DIO/CSo) per unit leaf area of wheat seedlings showed an overall trend of initially increasing and then decreasing. Among these, the energy trapped (TRO/CSo) and transferred (ETO/CSo) peaked at a DLI of 32.4 mol m^−2^ d^−1^ (Figure S1). Further analysis of OJIP fluorescence kinetics indicated that both V_J_ and V_I_ initially decreased and then stabilized with the increasing DLI, suggesting that an appropriate DLI can enhance the functional integrity of the PSII donor–acceptor system in wheat seedling leaves. Increasing the DLI to 39.6 mol m^−2^ d^−1^ effectively enhanced the activity of the oxygen-evolving complex (OEC) while also increasing the terminal electron sink capacity of PSI (as indicated by the appearance of the L-band and a significant decrease in FL/FJ), thereby promoting the development of the photosynthetic apparatus (Figures S2–S4). These results demonstrate that an appropriate DLI improved the development of the photosynthetic apparatus in wheat seedling leaves. However, when the DLI exceeds 39.6 mol m^−2^ d^−1^, higher light likely induces photo-inhibition, limiting the development of photosynthetic structures.

### 2.6. Days to Jointing, Heading, and Flowering in Wheat After Transplanting

**Figure 6:**
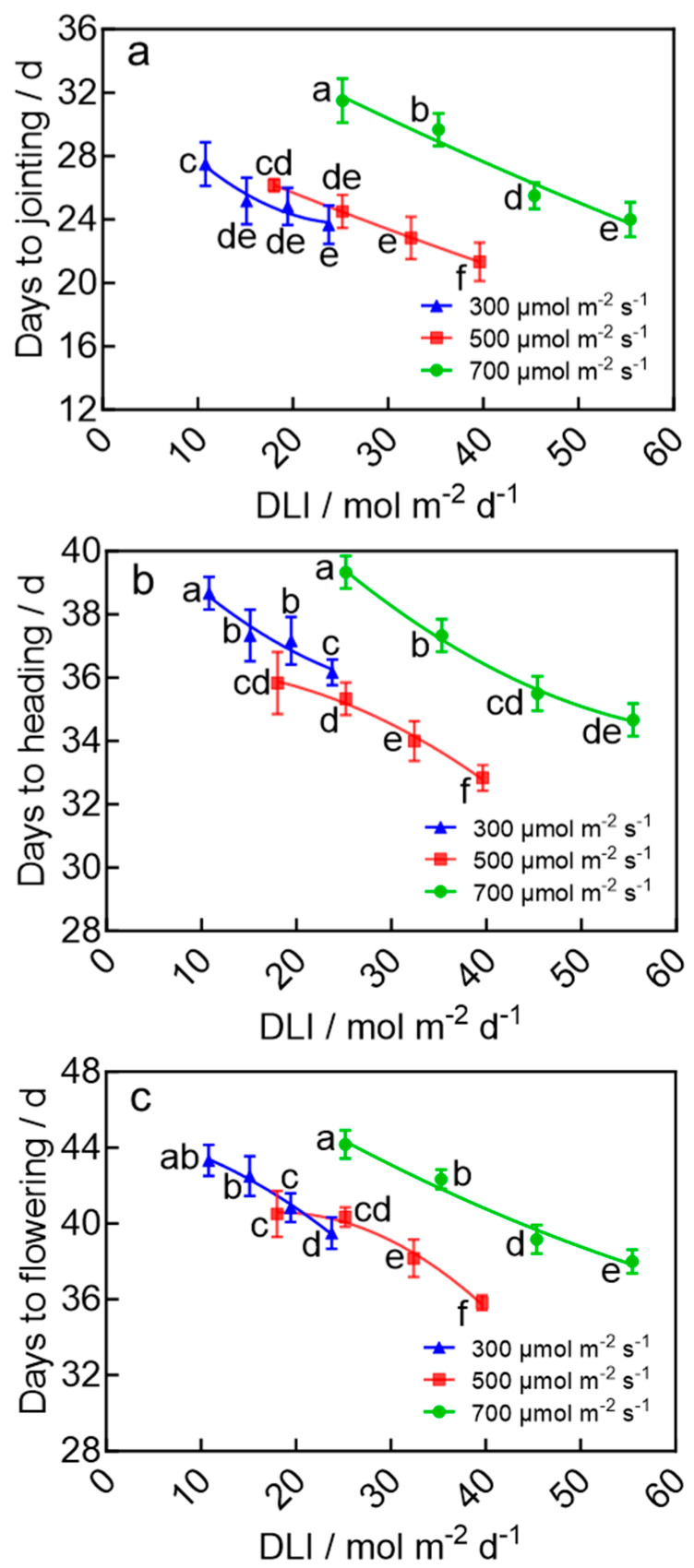
Days to jointing (**a**), heading (**b**), and flowering (**c**) of wheat plants after transplanting in response to the seedling-stage DLI under the LED plant factory. The four data points on each curve of the same color correspond to photoperiods of 10, 14, 18, and 22 h d^−1^, respectively, under the same light intensity. Different letters for the same parameter indicate significant differences (*p* < 0.05), according to Duncan’s multiple range test.

In contrast to the DLI, the photoperiod during the seedling stage exhibited a more pronounced accelerating effect on the days to jointing, heading, and flowering of wheat plants. Under the same light intensity, extending the photoperiod significantly shortened the days to jointing, heading, and flowering after transplanting, with the shortest durations consistently observed under a photoperiod of 22 h d^−1^ (Figure 6). However, an optimal DLI was still critical. When seedlings were grown under a DLI of 39.6 mol m^−2^ d^−1^, the days to jointing, heading, and flowering occurred the earliest, at 21.3 d, 32.8 d, and 35.8 d, respectively—all significantly sooner than those under other DLI treatments. These results indicate that combining an extended photoperiod with the optimal DLI during the seedling stage can effectively shorten the transition from vegetative to reproductive growth and accelerate the heading and flowering processes in wheat after transplanting.

## 3. Discussion

### 3.1. Effect of DLI on Growth and Photosystem Performance of Wheat Seedlings

The light environment has a profound influence on plant morphogenesis, physiology, growth, and crop quality [23,24,25]. The DLI, which represents the total amount of light received per day, provides a comprehensive measure of the plant’s light environment since it integrates both light intensity and photoperiod [26]. This study found that a DLI of 39.6 mol m^−2^ d^−1^ resulted in higher aboveground biomass accumulation, a maximum root-to-shoot ratio, and the highest seedling (Figure 2 and Figure 3). These parameters were significantly lower at DLIs below this point and did not significantly increase at higher DLIs. However, when the DLI exceeded 40 mol m^−2^ d^−1^, excess light energy was not efficiently utilized for carbon assimilation, which likely led to the accumulation of photo-oxidative stress under high light intensity and long days [27]. This stress, in turn, suppressed photosystem functionality and limited plant growth (Figure 3, Figure 4 and Figure 5). A similar growth response to the DLI was observed in many crops, and the optimal DLI was shown to be species-dependent [28,29,30]. For example, studies by Zhang et al. [30] and Yan et al. [28] also reported a linear relationship between the DLI and shoot/root dry and fresh weight in plants and demonstrated that increasing the DLI within a certain range accelerated plant growth, whereas beyond a threshold, growth stabilized or was inhibited [31]—consistent with the findings of this study. For wheat speed-breeding, a previous study recommended a DLI of 55.4 mol m^−2^ d^−1^ across the entire growth cycle or a DLI of 17.0 mol m^−2^ d^−1^ during the seedling stage, followed by a higher DLI for the rest of the growth cycle [32]. In this study, we further refined the recommendation DLI specifically for the seedling stage. By testing a wide range of DLIs, we recommend 39.6 mol m^−2^ d^−1^ as the optimal DLI for wheat seedlings.

The DLI also affected the development of photosynthetic machinery in wheat seedlings (Figure 4, Figure 5 and Figures S1–S4). The optimal DLI range of 35–40 mol m^−2^ d^−1^ resulted in a higher PI_abs_, an increased density of RC/CS, φEo, Sm, along with enhanced stability of the OEC and increased synthesis of photosynthetic pigments (Figure 4, Figure 5 and Figure S3). The enhanced photosynthetic machinery likely led to a more efficient photosynthetic process, which further promoted the growth of wheat seedlings. Moreover, previous research has shown that increasing the DLI not only improves the efficiency of the light reaction but also enhances the efficiency of the Calvin-Benson cycle by increasing Rubisco content [20]. Seedlings with this robust photosynthetic machinery can potentially assimilate more carbon and achieve faster growth and development after transplanting. Therefore, the robust photosynthetic machinery developed under the optimal DLI of 39.6 mol m^−2^ d^−1^ likely contributed to accelerated development and earlier flowering after transplanting.

Notably, plant responses to daily light integral (DLI) differed under high light intensity (700 μmol·m^−2^·s^−1^) compared to medium and low light intensities (500 and 300 μmol·m^−2^·s^−1^). Biomass accumulation under high light intensity combined with a short photoperiod was significantly lower than under medium light intensity with a long photoperiod (Figure 3). This discrepancy can be attributed to high light-induced photoinhibition, which markedly reduced the seedling leaves’ capacity for light energy absorption (ABS/CSo), capture (TRo/CSo), and dissipation (DIo/CSo) (Figure S1), thereby decreasing light use efficiency and restricting growth. Similar phenomena have been reported in other crops: under the same DLI, extending the photoperiod promoted lettuce growth by increasing chlorophyll content [33], while combining a longer photoperiod with reduced light intensity improved growth in lettuce and mizuna by enhancing light interception and photosystem II quantum yield [34].

### 3.2. Higher DLI Promotes Tillering and Root Biomass Accumulation of Wheat Seedlings

Tillering and root development, which established the foundation for spike number and nutrient uptake [35], responded to the DLI differently from shoot biomass. Both tiller number and root biomass increased continuously with the increasing DLI (Figure 1c and Figure 3b,d), consistent with previous studies on winter wheat [36]. Notably, even when a higher DLI induced photo-oxidative stress and suppressed shoot growth, tillering and root biomass continued to increase (Figure 1c and Figure 3d). This phenomenon suggests that light signals may regulate tillering and root growth independently from shoot growth. A high DLI potentially drives tillering through non-carbon pathways, such as a cytokinin-mediated axillary bud activation mechanism [36,37]. Likewise, under high light, plants adjust biomass allocation to prioritize root growth. This adaptive strategy enhances the plant’s capacity for water and nutrient uptake, allowing it to cope with high light stress [38,39].

### 3.3. Photoperiod and DLI During the Seedling Stage Synergistically Accelerate Heading and Flowering in Wheat

The rapid transition from vegetative to reproductive growth is a core physiological process in establishing rapid breeding systems for wheat. This study demonstrated that extending photoperiod and optimizing the DLI during the seedling stage synergistically reduce the time to jointing, heading, and flowering after transplanting. Under a DLI of 39.6 mol m^−2^ d^−1^ and a photoperiod of 22 h d^−1^, the time from sowing to flowering was only 35.8 days—a significant reduction of 17.3% or 7.5 days compared to the treatment with a DLI of 10.8 mol m^−2^ d^−1^ and a photoperiod of 10 h d^−1^ (Figure 6). In this study, the prolonged photoperiod of 22 h during the seedling stage likely upregulated the expression of wheat *FLOWERING LOCUS T* (*TaFT1*) gene and induced the production of florigen protein (TaFT1) in leaves [40]. The florigen protein was transported through phloem to the shoot apical meristem (SAM) [41], where it initiated irreversible commitment to flowering even before transplanting [42,43,44]. Additionally, the accelerated reproductive transition following optimized the seedling-stage DLI (39.6 mol m^−2^ d^−1^) of the same photoperiod may be further attributed to the better morphology, higher shoot biomass accumulation, and enhanced photosystem development (Figure 1, Figure 2, Figure 3 and Figures S1–S4). These seedlings are likely to capture and utilize light more efficiently after transplanting than seedlings from other DLI treatments, ultimately achieving faster growth and development.

## 4. Materials and Methods

### 4.1. Plant Materials and Growth Conditions

**Figure 7:**
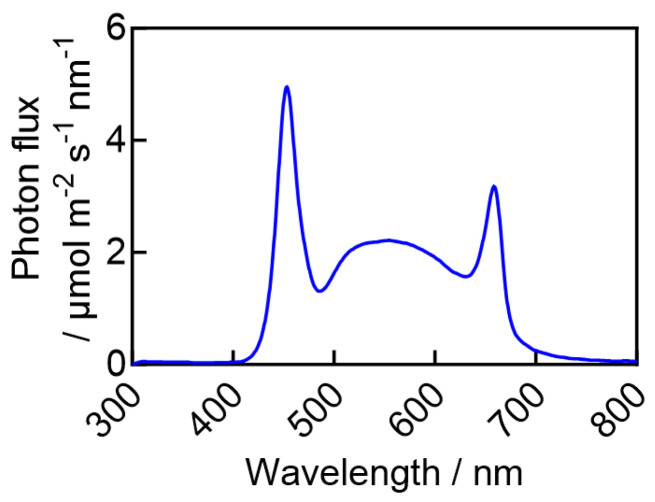
Spectral distribution in the LED light environment experimental zone.

The experiment was conducted in an LED plant factory located at China Agricultural University (40°0′ N, 116°21′ E). Spring wheat (cv. Ningchun 4) was used as plant material. Plump and uniform seeds were selected and soaked in water for 12 h at an initial temperature of 65 °C, allowing the water to cool naturally to room temperature. They were then placed in Petri dishes lined with moist filter paper for germination in the dark at 25 ± 1 °C for 24 h. Seeds with consistent germination were transplanted to 32-cell trays (L54 cm × W28 cm × H5 cm) filled with a peat: vermiculite: perlite mixture (3:1:1, *v*/*v*/*v*), with one seed per cell. After 2 days of dark incubation, all experimental zones were illuminated with white LED lights (model W-LED-4000K, Beijing Shengyanggu Technology Co., Ltd., Beijing, China) with a red-to-blue ratio of 1.6. The spectral distribution is presented in Figure 7, and tests confirmed that it remained consistent across all experimental zones. To ensure uniform light distribution in each unit, LEDs were arranged with irregular horizontal spacing. Throughout the cultivation period, the temperature, relative humidity, and CO_2_ concentration were maintained at 22 ± 1 °C, 70% ± 5%, and 800 ± 50 μmol mol^−1^, respectively. Seedlings were placed in an ebb-and-flow system, where each fertigation event lasted 30 min. The nutrient solution was based on the Enshi nutrient solution (Japanese garden test formulation, in mg L^−1^): KNO_3_ 808.0, Ca(NO_3_)_2_ 944.0, MgSO_4_ 492.0, NH_4_H_2_PO_4_ 152.0, EDTA-Fe 30.0, H_3_BO_3_ 2.86, MnSO_4_·4H_2_O 2.13, ZnSO_4_·7H_2_O 0.22, CuSO_4_·5H_2_O 0.08, (NH_4_)_6_Mo_7_O_24_·4H_2_O 0.02. The pH was adjusted to 5.6–6.0, and electrical conductivity (EC) was maintained at 2.0–2.4 mS cm^−1^. Seedlings received one fertigation application with half-strength nutrient solution after emergence. After the second true leaf expanded, a regular fertigation schedule with a full-strength solution was initiated every 2 days.

### 4.2. Light Treatments

Three cultivation racks (L 1.25 m × W 0.60 m × H 2.10 m) were set up in an LED plant factory. Each rack has four levels, with a height of 0.5 m per level. Each level holds four 32-cell trays. Twelve DLI levels (ranging from 10.8 to 55.4 mol m^−2^ d^−1^) were tested by combining three light intensities (300, 500, and 700 μmol m^−2^ s^−1^) with four photoperiods (10, 14, 18, and 22 h d^−1^) (Table 1) to investigate their effects on wheat seedlings. The 12 DLI treatments were randomly distributed across the three racks, which represent one full replicate of this study. The light treatments last for 12 days, during which seedling growth, morphological, and physiological parameters were measured.

Additionally, eight representative seedlings from each experimental unit were randomly selected and transplanted into 6.28 L plastic pots (20 cm × 20 cm) for continued cultivation under uniform conditions until flowering. Environmental parameters were as follows: light intensity 700 ± 20 μmol m^−2^ s^−1^, photoperiod 22 h d^−1^, temperature 22 ± 1 °C, relative humidity 70% ± 5%, and CO_2_ concentration 800 ± 50 μmol mol^−1^. An automated irrigation system was used to supply 240 mL of nutrient solution per pot per day, with a drainage volume of 72 mL per pot. Wheat plants were fertigated with full-strength Enshi nutrient solution as described above. The dates of jointing, heading, and flowering were recorded. The experiment was replicated three times in a completely randomized block design, with time as the blocking factor.

### 4.3. Morphology and Quality of Wheat Seedlings

Five seedlings were randomly selected from each experimental unit for measurements of main culm leaf number, tiller number, plant height, stem diameter, number of secondary roots, and biomass. Plant height was measured using a ruler. The stem diameter was determined using a digital caliper. Shoots and roots were separated, and their fresh weights were measured using a centigram balance. Shoot and root tissues were then oven-dried at 105 °C for 30 min to deactivate enzymes, followed by drying at 75 °C for 72 h until constant weight was achieved. Shoot and root dry weights were measured using a precision balance (FA1204B, Shanghai Precision & Scientific Instrument Co., Ltd., Shanghai, China). The seedling index and root-to-shoot ratio were calculated as follows:

(1) $$Seedling\;index=\left(\frac{Stem\;diameter}{Plant\;height}+\frac{Root\;dry\;weight}{Shoot\;dry\;weight}\right)\times Plant\;dry\;weight$$

(2) $$Root-to-shoot\;ratio=\frac{Root\;dry\;weight}{Shoot\;dry\;weight}$$

### 4.4. Leaf Chlorophyll Contents and Chlorophyll Fluorescence of Wheat Seedlings

Five seedlings were randomly selected from each experimental unit. The first three fully expanded leaves on the main culm were excised, chopped, and combined into a single sample. Approximately 0.1 g of tissue was soaked in 10 mL of 80% acetone and kept in the dark at room temperature for 48 h or until completely bleached. The extract was agitated, and 3 mL was transferred to a spectrophotometer cuvette. Absorbance was measured at 663 nm, 645 nm, and 470 nm using a spectrophotometer (UV-3150, Shimadzu Corporation, Kyoto, Japan). Chlorophyll a, chlorophyll b, and carotenoid concentrations were calculated according to the equations [45,46]:

(3) $$Chl\;a=\frac{\left(12.7\times A663-2.69\times A645\right)\times 0.001V}{W}$$

(4) $$Chl\;b=\frac{\left(22.9\times A645-4.86\times A663\right)\times 0.001V}{W}$$

(5) $$Car=\frac{\left(1000\times A470-3.27\times A663-104\times A645\right)\times 0.001V}{229W}$$

where *Chl a*, *Chl b*, and *Car* represent the contents of chlorophyll a, chlorophyll b, and carotenoids (mg g^−1^), respectively; A663, A645, and A470 represent the sample absorbance at 663 nm, 645 nm, and 470 nm, respectively; *V* is the volume of the extraction solution (10 mL); and *W* is the fresh weight of the sample (g).

Seven seedlings were randomly selected from each experimental unit for chlorophyll fluorescence measurements. After 30 min of dark adaptation, the rapid chlorophyll fluorescence induction kinetics (OJIP curve) of the third leaf on the main culm were measured using a Multi-Function Plant Efficiency Analyzer (M-PEA, Hansatech Instruments Ltd., King’s Lynn, UK). The obtained OJIP curves were subsequently analyzed using the JIP-test protocol [47,48] or according to the M-PEA manual. The points O, K, J, I, and P on the OJIP curve correspond to the instantaneous fluorescence intensities observed at 0.01, 0.3, 2, 30, and 1000 ms, respectively, labeled as Fo, Fk, FJ, FI, and Fm. Vt denotes the relative variable fluorescence at time t, calculated as Vt = (Ft − Fo)/(Fm − Fo). W_OJ_ represents the normalized fluorescence for the O–J phase, calculated as W_OJ_ = (Ft − Fo)/(FJ − Fo). W_OI_ is the normalized fluorescence for the O–I phase, calculated as W_OI_ = (Ft − Fo)/(FI − Fo), reflecting the change in variable fluorescence between points O and I. The JIP-test parameter W_K_ = (Fk − Fo)/(FJ − Fo) indicates the variable fluorescence intensity at the K-step relative to the amplitude FJ − Fo.

### 4.5. Statistical Analysis

Figures were plotted using GraphPad Prism 10. Data are shown as mean ± standard deviation. Differences among treatment groups were assessed using one-way ANOVA followed by Duncan’s multiple range test in SPSS 21.0 software (α = 0.05). Means noted with the same letter are not significantly different, while different letters indicate significant differences.

## 5. Conclusions

This study identified that an optimal DLI of 39.6 mol m^−2^ d^−1^ (using a light intensity of 500 μmol m^−2^ s^−1^ and a 22 h d^−1^ photoperiod) produced high-quality wheat seedlings, resulting in higher shoot biomass accumulation, peak values in the seedling index and root-to-shoot ratio, and enhanced photosystem performance. Furthermore, the optimal DLI, coupled with an extended photoperiod (22 h d^−1^) during the seedling stage, synergistically accelerated jointing, heading, and flowering after transplanting. This research provides a quantitative and effective lighting strategy for advancing speed-breeding systems of wheat.

## Figures and Tables

**Table 1:** Different daily light integral treatments for wheat seedlings.

Treatments	Light Intensity	Photoperiod	DLI
	μmol m^−2^ s^−1^	h d^−1^	mol m^−2^ d^−1^
P300-H10	300	10	10.8
P300-H14		14	15.1
P300-H18		18	19.4
P300-H22		22	23.8
P500-H10	500	10	18.0
P500-H14		14	25.2
P500-H18		18	32.4
P500-H22		22	39.6
P700-H10	700	10	25.2
P700-H14		14	35.3
P700-H18		18	45.4
P700-H22		22	55.4

## Data Availability

The original contributions presented in this study are included in the article/Supplementary Materials. Further inquiries can be directed to the corresponding authors.

## Supplementary Materials

The following supporting information can be downloaded at https://www.mdpi.com/article/10.3390/plants15020326/s1, Figure S1: ABS/CSo (a), TRo/CSo (b), Eto/CSo (c), and DIo/CSo (d) of wheat seedling leaves in response to DLI under the LED plant factory; Figure S2: Analysis of OJIP fluorescence kinetics in wheat seedling leaf; Figure S3: Analysis of oxygen-evolving complex (OEC) function in wheat seedling leaf based on OJIP fluorescence kinetics and Figure S4: Analysis of PSI acceptor side function in wheat seedling leaf based on OJIP fluorescence kinetics.
