# Supplementary material for: Optimizing Daily Light Integral in Seedling Stage Accelerates Heading and Flowering in Wheat Under LED Lighting
Source: Plants (Basel). 2026 Jan 21;15(2):326. doi: 10.3390/plants15020326 (PMC12844831; doi:10.3390/plants15020326)
Supplement: Supplementary file 1 [file plants-15-00326-s001.zip › plants-4081393-supplementary.pdf]

Supplementary Material

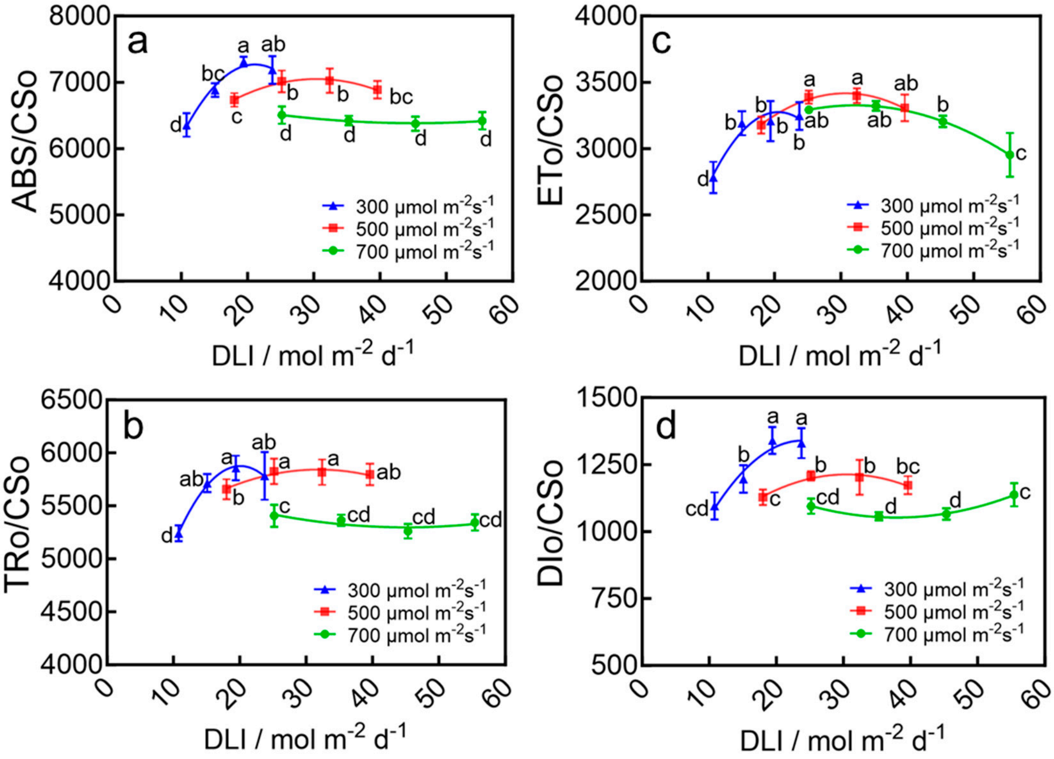

Figure S1. ABS/CSo (a), TRo/CSo (b), Eto/CSo (c), and DIo/CSo (d) of wheat seedling leaves in response to DLI under the LED plant factory. Different letters for the same parameter indicate significant differences ($p < 0.05$), according to Duncan's multiple range test.

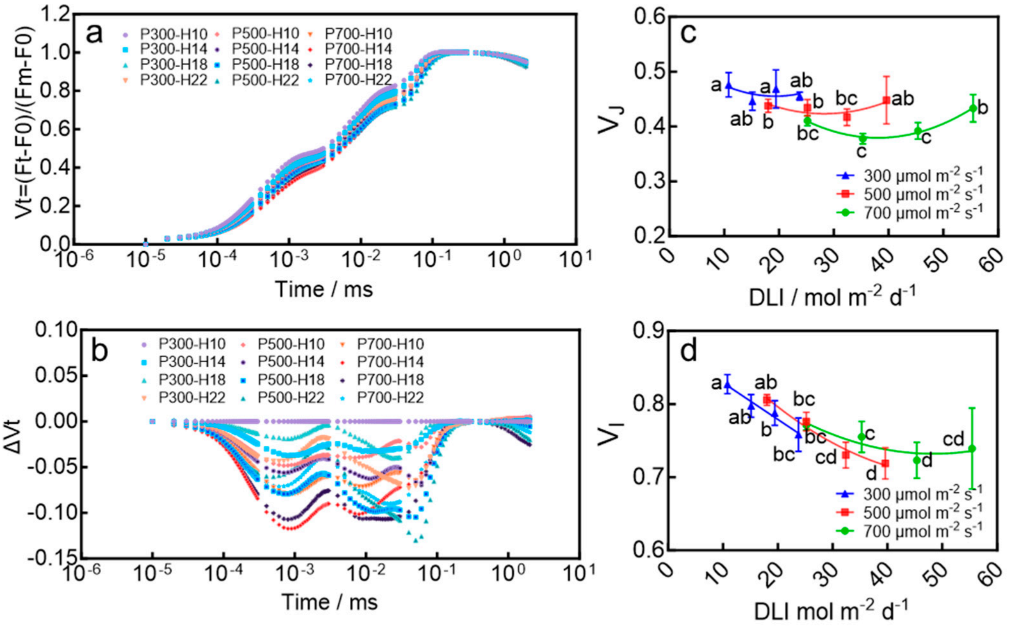

Figure S2. Analysis of OJIP fluorescence kinetics in wheat seedling leaf. Chlorophyll fluorescence parameter Vt under different treatments was normalized to obtain ΔVt, followed by analysis of the chlorophyll fluorescence values V_J and V_I corresponding to the J-step and I-step, in order to investigate

the effect of DLI on the functional integrity of the donor–acceptor side of PSII in wheat seedling leaves. Different letters for the same parameter indicate significant differences ($p < 0.05$), according to Duncan's multiple range test.

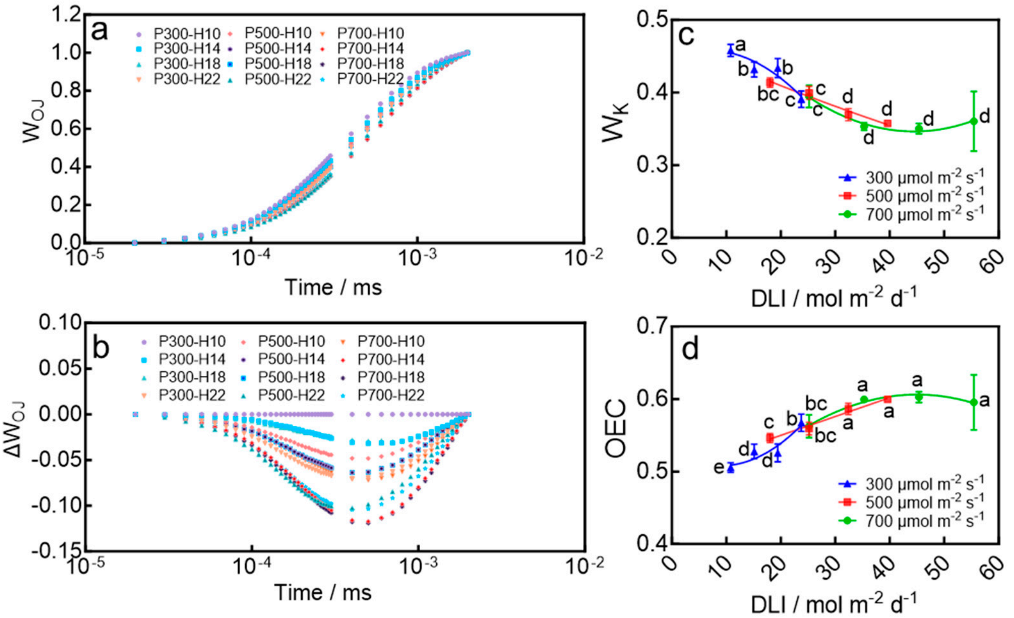

Figure S3. Analysis of oxygen-evolving complex (OEC) function in wheat seedling leaf based on OJIP fluorescence kinetics. The O-J phase of the OJIP curve was normalized to yield WOJ. Using the P300-H10 treatment as the control, the chlorophyll fluorescence kinetic difference ΔWOJ was calculated to detect the appearance of the K-band, which indicates inactivation of the oxygen-evolving complex (OEC) on the donor side of PSII. Subsequently, Wk and OEC status were further analyzed to investigate the effects of DLI on OEC function at the PSII donor side in wheat seedling leaves. Different letters for the same parameter indicate significant differences ($p < 0.05$), according to Duncan's multiple range test.

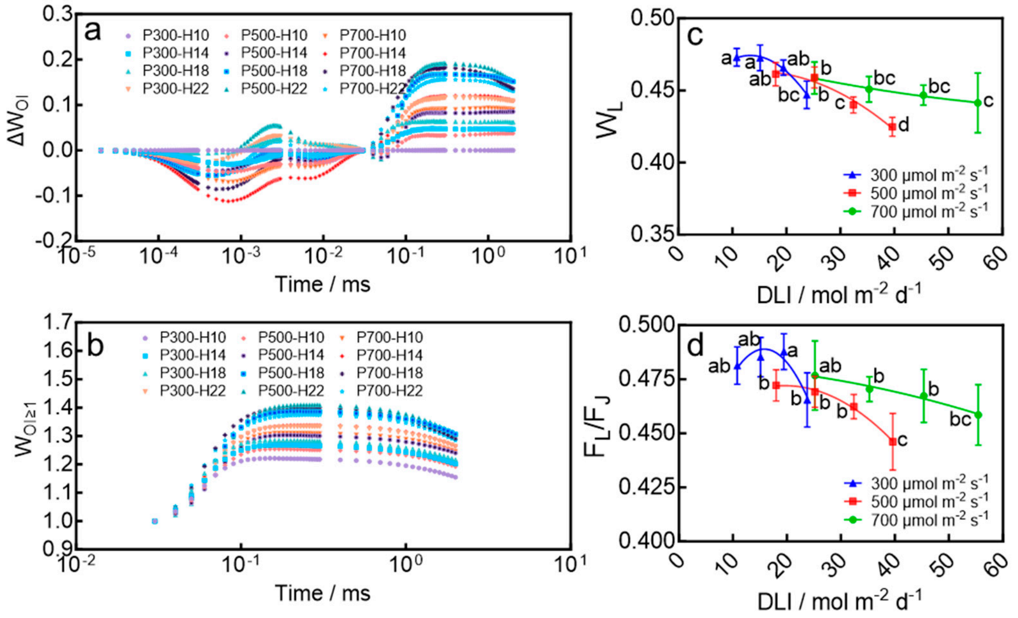

Figure S4. Analysis of PSI acceptor side function in wheat seedling leaf based on OJIP fluorescence kinetics. The O-I phase of the OJIP curve was normalized to obtain WOI, and ΔWOI was calculated using P300-H10 as the control. ΔWOI ⩾ 1 was used to determine the appearance of the L-band. Further analysis of the mechanism underlying changes in the L-band was conducted by combining the WL parameter, which reflects the electron transport bottleneck, and the FL/FJ ratio, which indicates the risk of over-reduction on the acceptor side of PSI. The L-band reflects the aggregation among different components of PSII or the energy transfer connectivity between antenna pigments and PSII active reaction centers (RCs). If WL and ΔWL increase significantly while FL/FJ shows no significant change, it indicates that the rise in the L-band is mainly due to an increase in the J-phase. If FL/FJ increases significantly, it suggests that the treatment induces the appearance of the L-band by reducing the aggregation or energy connectivity among PSII components. Based on this analysis, the effect of DLI on the terminal electron pool capacity of PSI in wheat seedling leaves was further investigated. Different letters for the same parameter indicate significant differences ($p < 0.05$), according to Duncan's multiple range test.
